# Microplastics and IgA nephropathy: a novel environmental hypothesis of immune dysregulation

**DOI:** 10.1080/0886022X.2026.2735540

**Published:** 2026-09-30

**Authors:** Joel Shah, William Wells-Gatnik, Biff F. Palmer, Deborah J. Clegg

**Affiliations:** ^a^ Department of Internal Medicine, Texas Tech University Health Sciences Center, El Paso, TX, USA; ^b^ Departments of Education and Internal Medicine, Texas Tech University Health Sciences Center, El Paso, TX, USA; ^c^ Department of Internal Medicine, Vice President for Research, Texas Tech University Health Sciences Center, El Paso, TX, USA

**Keywords:** IgA nephropathy, microplastics, nanoplastics, galactose deficient IgA1 antibodies

## Abstract

IgA nephropathy (IgAN) is the most common glomerulonephritis worldwide, characterized by a 4-hit pathogenesis primarily driven by mucosal immune dysregulation in the gut-associated lymphoid tissue (GALT). However, the specific environmental triggers initiating this cascade remain elusive. We propose a novel hypothesis that chronic exposure to ubiquitous micro- and nano-plastics (MNPs) serves as a primary environmental catalyst for IgAN. Through a proposed 5-step cascade, we outline how ingested MNPs directly degrade intestinal tight junctions, precipitating a ‘leaky gut’ that permits the synergistic influx of plastics and commensal bacterial antigens into the GALT. Acting as a ‘Trojan horse,’ MNP-corona complexes evade immune detection but trigger chronic inflammation and B-cell dysregulation upon internalization by phagocytes. This persistent immune stimulation drives the aberrant production of galactose-deficient IgA1. Furthermore, we hypothesize that circulating MNPs co-deposit in the renal mesangium alongside immune complexes, acting as a dual generator of both systemic immune complexes and direct structural kidney injury. This potential gene-environment interaction is supported by the intersecting high prevalence of environmental MNPs and IgAN in Asia. Finally, we delineate future *in vivo*, observational, and epidemiological investigations required to establish this pathophysiological link.

## Introduction

Worldwide, IgA nephropathy represents the most common glomerulonephritis [1]. IgA nephropathy pathogenesis follows a 4-hit hypothesis characterized by 1. aberrant B-cell production of galactose-deficient IgA1 antibodies (Gd-IgA1), 2. formation of anti-glycan immune complexes with circulating IgA/IgG, 3. deposition of immune complexes in the renal mesangium, and 4. inflammation and glomerulosclerosis resulting in the development of IgA nephropathy [2,3]. The primary driving mechanism associated with the 4-hit hypothesis of IgA nephropathy is mucosal immunity dysregulation in gut-associated lymphoid tissue (GALT). Though significant advances in mapping the pathogenesis of IgA nephropathy have been made, GALT-associated immune dysregulation is incompletely understood. Here we suggest there is evidence to support the hypothesis environmental exposures may be inducers of immune-mediated kidney diseases such as IgA nephropathy [4].

Micro and nano-plastics (MNP) represent an environmental threat to human health and there is a growing body of literature connecting MNP exposure to various pathologies spanning multiple organ systems. MNP are plastic particles generated from degraded plastic found in water, air, and food sources [5,6]. MNP enter the human body *via* dermal exposure, ingestion or inhalation and have been detected in the circulatory (blood) and gastrointestinal systems (stool) and nearly every human tissue [6]. MNP have been shown to cross the epithelium and promote immune dysregulation *via* interactions with immune cells *in vivo* and *in vitro* models [7,8].

We propose one hypothesis to explore, that chronic MNP exposure could be a novel environmental trigger of immune dysregulation in GALT, thus potentially precipitating the aberrant production of Gd-IgA1 and the development of IgA nephropathy in genetically susceptible individuals. Furthermore, we posit the possibility MNP may directly deposit within the glomerulus and act synergistically with immune complexes to precipitate structural kidney injury. We would like to emphasize that each mechanistic step outlined in this manuscript draws largely from MNP related literature that has not yet been examined within the context of gut-associated lymphoid tissue or in the context of IgA nephropathy. As such, the aim of our proposal is to present a testable framework for future research rather than assertion of an established pathway.

Microfold (M) cells surrounding Peyer’s patches may serve as a conduit for internalization of MNP into GALT. *In vivo* and *in vitro* studies have demonstrated upon internalization, MNP bind to various immunoglobulins, plasma, and complement proteins, forming a corona [8]. Multiple factors influence the composition and thickness of this corona, including but not limited to MNP polymer type, particle size, surface charge, hydrophobicity, degree of surface oxidation, alongside local biochemical conditions such as gut pH and bile salt composition [9]. The heterogeneity of the MNP-corona complex likely varies by source exposure and gastrointestinal environment given the variables associated with it. MNP-corona complexes have been shown to stimulate phagocytes, including lymphocytes, macrophages, and dendritic cells *via* immune recognition, marking the complexes for phagocytosis. This demonstrates a method by which MNPs may act as direct and persistent activators of the immune system though more literature is necessary to elucidate such a mechanism.

## MNP-induced intestinal permeability and ‘leaky gut’

Beyond passive absorption through M-cells, toxicological evidence from animal and *in vitro* models suggests that MNP exposure actively, physically, and chemically degrades intestinal tight junctions, such as the zonula occludens [10]. Disruption of the epithelial barrier by MNP may result in intestinal permeability, referred to as a ‘leaky gut.’ This structural compromise represents a potential critical catalyst in the pathogenesis of IgA nephropathy and may allow for the continuous influx of MNP, bacterial antigens, and luminal endotoxins to bypass mucosal defenses. Consequently, this leaky gut environment may force a synergistic hyper-activation of the underlying GALT, potentially setting the stage for chronic, autonomous immune dysregulation.

## The MNP ‘trojan horse’

MNP have been demonstrated to modulate immune function in the human body, including cytokine release, phagocyte stimulation, intracellular signaling pathway disruption, and oxidative stress [8]. The interaction between micro- and nanoplastics (MNPs) and biological fluids immediately results in the adsorption of surrounding biomolecules onto the particle surface, a phenomenon known as “protein corona” or biocorona formation. MNP-corona complexes increase signal recognition and uptake by lymphocytes, dendritic cells, and macrophages *via* the Fc receptor and have been shown to activate the NLRP3 inflammasome thus promoting IL-1β pro-inflammatory cytokine release [9,10]. Upon internalization, MNP complexes can inhibit intracellular signaling pathways leading to cellular adaptive stress responses, resulting in inflammation. Due to their formation with cellular proteins, MNP may evade detection by the immune system, allowing for their release into the systemic circulation and deposition into various organ systems. Yang et al. label this series of effects as a ‘Trojan horse’ mechanism due to the ability of MNP to internalize and promote pathological adaptive responses intracellularly while evading immune system surveillance [8]. Additionally, the chronicity of MNP exposure has been proposed to have effects on B-cell class switching [11]. We hypothesize that an MNP induced proinflammatory response *via* accumulation within GALT may contribute to B-cell class switching dysregulation and the potential formation of Gd-IgA1. Whether this proposed mechanism preferentially favors Gd-IgA1 accumulation as opposed to other immunoglobulin isotypes has not yet been demonstrated in the literature.

## Summary of potential mechanisms by which MNP may influence IgA nephropathy

To synthesize these immunological principles, we present a 5^th^ component to the 4-hit mechanism by which MNP may contribute to the formation of IgA nephropathy (Figure 1). As opposed to established hits, MNP exposure is largely ubiquitous across the general population. Thus, we propose MNP acts primarily as an environmental modifier or amplifier that acts within genetically susceptible hosts rather than as an independent disease defining hit. This proposed cascade outlines the journey of MNPs from initial environmental exposure, to compromising endothelial barriers, to sequential disruption of mucosal and systemic immunity, culminating in direct renal injury (Figure 1).

**Figure 1.**
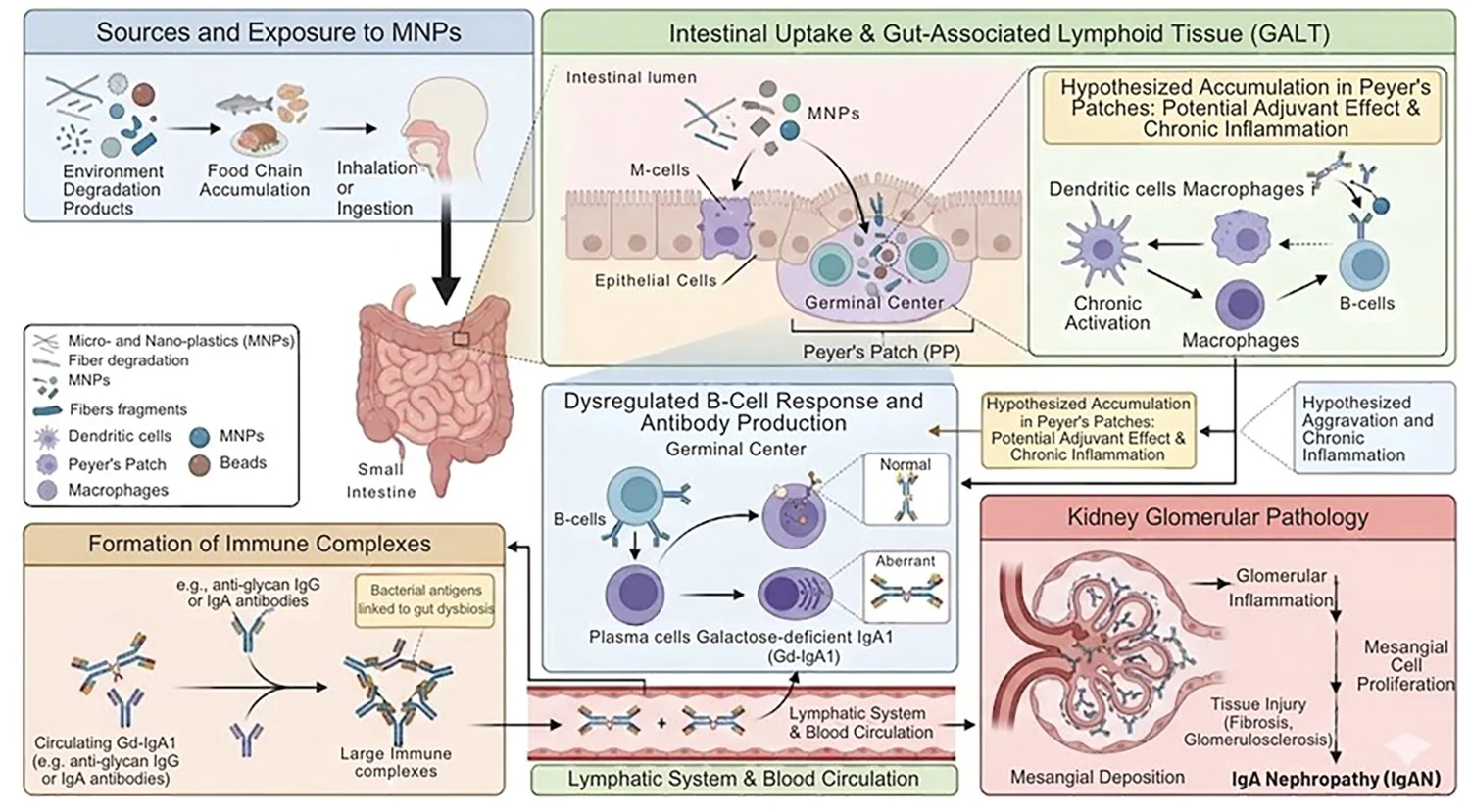
Proposed hypothesis linking MNPs to the development of IgA nephropathy. Environmental degradation products are consumed and absorbed in the small intestine by a variety of cells, including Peyer’s Patches. The accumulation of MNPs in Peyer’s Patches leads to chronic inflammation *via* activation of B-cells from dendritic and macrophage activation, leading to a cycle of chronic inflammation. Chronic B-cell activation may induce dysregulation with incomplete class switching, resulting in the production of galactose-deficient IgA1 (Gd-IgA1), which forms immune complexes with anti-glycan IgG or IgA with amplification by gut dysbiosis from circulating bacterial antigens. The resultant large immune complexes circulate in the blood and lymphatic system before depositing in the mesangium within the renal glomerulus. This, in turn, promotes glomerular inflammation and responsive mesangial cell proliferation, leading to tissue injury such as fibrosis, culminating in the development of IgA nephropathy.

### Uptake and epithelial barrier compromise

MNP are ubiquitous in the environment and can influence human health through ingestion or inhalation and transportation to the blood *via* absorption through M-cells in Peyer’s Patches of the small intestine. MNP may directly compromise tight intestinal junctions precipitating epithelial permeability [11]. Barrier disruption in turn potentially allows for an influx of both MNP and various bacterial antigens into GALT promoting gut dysbiosis.

### Immune cell activation and phagocytosis

The gradual accumulation of MNP in Peyer’s patches and the formation of a corona can stimulate a proinflammatory state characterized by Fc-receptor mediated internalization by macrophages, lymphocytes, and dendritic cells [9,10]. Alongside MNP-corona complexes, bacterial antigens can synergistically trigger the innate immune system *via* toll-like receptor (TLR) activation and the NLRP3 inflammasome, amplifying mucosal immune responses.

### B-Cell dysregulation and alteration of class switching

A sustained chronic inflammatory state, in turn, would be expected to cause B-cell dysregulation and altered antibody production. MNP induced immune activity has been proposed to stimulate the overproduction of various cytokines including B-cell activating factor (BAFF), a proliferation-inducing ligand (APRIL), and IL-6. IL-6 has been shown to independently regulate activity and expression of core 1 B1,3-galactosyltransferase (C1GalT1) and its molecular chaperone Cosmc while upregulating a2,6-sialytransferase (ST6GalNAc-II) which together favors production of galactose-deficient IgA1 sialylated (Gd-IgA1) [12]. The release of BAFF and APRIL can drive T-cell-independent B-cell class switching which, in combination with IL-6 downstream effects, favors production of Gd-IgA1 [12]. We propose that MNP-driven cytokine dysregulation, amplified bacterial gut dysbiosis, may disrupt glycosyltransferase effects in IgA1 secreting cells providing a mechanistic bridge between MNP induced inflammation and GD-IgA1 production. This pathway has not yet been studied however and thus far represents an experimental gap in literature regarding MNP.

### Immune complex formation and deposition onto the renal mesangium

Aberrantly produced Gd-IgA1 possesses altered structural antigenicity and is therefore recognized as an autoantigen by the systemic immune system. Gd-IgA1 binds to circulating IgA or IgG, forming immune complexes which circulate through the lymphatic system and the blood before depositing on the renal mesangium. We hypothesize that MNP, based on their size and potential altered biochemical properties of these macromolecular complexes, could evade normal clearance, facilitating MNP entrapment and accumulation within the glomerulus.

### Development of IgA nephropathy and direct mesangial toxicity

Deposited mesangial immune complexes precipitate glomerular inflammation. Mesangial cells proliferate in response, causing further tissue injury including fibrosis, scarring, and glomerulosclerosis. Due to the proposed ‘trojan horse’ effect in which MNP evade immune detection, we hypothesize that they enter renal circulation before direct deposition into the renal mesangium thus playing a dual role as both a contributor to immune complex formation and as a source of direct injury. Here, they co-deposit with immune complexes, playing a dual role as both a generator of immune complexes and a source of direct structural injury to the kidney. If confirmed, deposition of both circulating Gd-IgA1-IgA/IgG immune complexes and MNP in the renal mesangium would precipitate IgA nephropathy pathogenesis and synergistically promote oxidative stress.

### IgA nephropathy in Asia, genetic predisposition, MNP exposure, or both?

Asia serves as the continent with the highest worldwide prevalence of IgA nephropathy [1]. Genome wide studies have identified population specific susceptibility loci that largely account for this geographic variation with significant genetic contributions [1,4,13]. Environmental MNP exposure and pollution burden may be assumed to correspond with regional plastic production, however, this relationship is not uniform. China and Japan, two countries with high IgA nephropathy prevalence, differ significantly in their per-capita plastic waste generation, industrial pollution burden, and fraction of mismanaged waste [14]. Japan generates comparable or greater per-capita plastic waste than China but is far more effective in waste management and thus has a lower environmental leakage [14]. This difference creates caution against corresponding direct MNP exposure with disease relationship *via* regional prevalence data alone. Therefore, we propose that MNP exposure may act as one of several environmental modifiers interacting with individuals with underlying genetic susceptibility and potentially amplifying disease burden thus contributing to the prevalence of IgA nephropathy in Asia as a secondary rather than primary factor. Whether MNPs contribute specifically to the interaction between genetics and the environment in IgA nephropathy remains a testable question that requires direct exposure measurements rather than simple correlation *via* ecological comparison.

## Missing link and future directions

The pathological mechanism by which MNP precipitate B-cell dysregulation and chronic inflammation has been established, however, to date no literature has analyzed its downstream effects on GALT or the production of Gd-IgA1 antibodies within the context of IgA nephropathy in humans. Clinical investigations are required to establish whether a pathophysiologic link exists between MNP and IgA nephropathy. Future *in vivo* human organoid studies exploring if there is a direct link between MNP serum Gd-IgA1 levels and the degree of mesangial deposition would be informative. Measurements of MNP exposure correlated to BAFF, APRIL, and IL-6 activity or accumulation in GALT with subsequent measurements of Gd-IgA1 levels and mesangial immune complex deposition is necessary to establish a mechanistic link though such correlative data alone would not establish causality.

Observational studies comparing blood and stool MNP burden in patients with newly diagnosed IgA nephropathy and other GALT-associated immune diseases versus healthy controls will also help determine if an observable association exists. Additionally, the use of specialized spectroscopy on renal biopsy samples may help to screen for MNP in patients with IgA nephropathy thus clarifying whether MNPs deposit in the renal mesangium alongside immune complexes of Gd-IgA1-IgA/IgG. Lastly, large-scale epidemiological studies should focus on the large prevalence of IgA nephropathy in Asia and examine if an association between genetic predisposition loci and measurements of MNP exposure exists [14]. The possibility of an environmental-gene interaction may help to identify if MNP help precipitate the disease directly or act as an environmental amplifier to a specific genetic locus.

## Conclusion

Though IgA nephropathy is known to be characterized by dysregulated mucosal immunity, the triggers involved in its pathogenesis are incompletely understood. MNP are increasingly more ubiquitous in the environment, with current toxicological evidence indicating they produce increased exposure and immunomodulatory effects in GALT function, though this evidence has not yet been specifically studied within the context of IgA nephropathy. We propose the hypothesis that MNP exposure may serve as a persistent stimulus in GALT, leading to aberrant Gd-IgA1 production through B-cell dysregulation, promoting IgA nephropathy pathogenesis. No empiric evidence exists in the literature thus far linking MNP to IgA nephropathy, and we therefore posit our hypothesis as a testable framework rather than as an established mechanism. Though more research is necessary to confirm the concept, we hope this hypothesis serves as an avenue for further investigations linking environmental pollutants to IgA nephropathy.

## Data Availability

The authors confirm that the data supporting the findings of this study are available within the article [and/or] its supplementary materials.
